# β-Adrenoceptor blockade modulates fusiform gyrus activity to black versus white faces

**DOI:** 10.1007/s00213-015-3929-7

**Published:** 2015-04-22

**Authors:** S. Terbeck, G. Kahane, S. McTavish, R. McCutcheon, M. Hewstone, J. Savulescu, L. P. Chesterman, P. J. Cowen, R. Norbury

**Affiliations:** Department of Psychology, University of Plymouth, Drake Circus, Plymouth, PL48AA UK; Oxford Centre for Neuroethics, University of Oxford, Littlegate House, St Ebbes St, Oxford, OX1 1PT UK; Department of Psychiatry, Warneford Hospital, Oxford, OX3 7JX UK; Department of Experimental Psychology, University of Oxford, South Parks Road, Oxford, OX1 2UD UK; The Ansel Clinic Nottingham, Clifton Lane Clifton, Nottingham, NG11 8NB UK; Department of Psychology, University of Roehampton, London, SW15 4JD UK

**Keywords:** Emotion, fMRI

## Abstract

**Introduction:**

The beta-adrenoceptor antagonist propranolol is known to reduce peripheral and central activity of noradrenaline. A recent study found that intervention with propranolol diminished negative implicit racial bias.

**Materials and method:**

The current study used functional magnetic resonance imaging (fMRI) in order to determine the neural correlates of this effect. Healthy volunteers (*N* = 40) of white ethnic origin received a single oral dose (40 mg) of propranolol, in a randomised, double-blind, parallel group, placebo-controlled design, before viewing unfamiliar faces of same and other race.

**Results and discussion:**

We found significantly reduced activity in the fusiform gyrus and thalamus following propranolol to out-group faces only. Additionally, propranolol lowered the implicit attitude score, without affecting explicit prejudice measure.

**Conclusion:**

These findings suggest that noradrenaline pathways might modulate racial bias by acting on the processing of categorisation in the fusiform gyrus.

## Introduction

Over the past decade, important advances have been made in understanding the neuropsychological mechanisms underlying racial biases. Research has suggested that social attitudes might have distinct components. In particular, this research has distinguished between an explicit component, an automatic/ implicit component and a behavioural component (Nosek et al. 2011). It has been further suggested that explicit aspects of intergroup attitudes are primarily cognitive, whereas implicit biases might have a stronger emotional component (Nosek et al. 2011). The role of emotion in implicit in-group bias has been demonstrated by recent research, which has also implicated key brain regions, such as the amygdala, the dorsolateral prefrontal cortex and the fusiform gyrus, in such implicit responses (Cunningham et al. 2004; Phelps et al. 2000; Stanley et al. 2008).

In line with this work, we recently found that a single dose of the β-adrenoceptor antagonist propranolol significantly reduced implicit racial bias as measured by the Implicit Association Test (IAT), but had no effect on explicit prejudice (Terbeck et al. 2012). This result suggests that noradrenergic-mediated arousal, which is sensitive to propranolol, may play a causal role in some forms of implicit racial bias. The reduction may have been associated with a modulation of central noradrenergic transmission in limbic and other brain areas, previously found to be activated by presentation of out-group faces. These areas include a network of limbic and cortical regions, including the amygdala, insula, fusiform gyrus, anterior cingulate cortex, and dorsolateral prefrontal cortex (Cunningham et al. 2004; Knutson et al. 2007; Phelps et al. 2000; Richeson et al. 2003; Stanley et al. 2008). For instance, previous studies have found differential activation to in- versus out-group faces in the amygdala (Phelps et al. 2000), the prefrontal cortex (Cunningham et al. 2004; Knutson et al. 2007; Richeson et al. 2003; Stanley et al. 2008), as well as the fusiform gyrus (Brosch et al. 2012; Contreras et al. 2013; Golby et al. 2001; Natu et al. 2011). These brain regions also show high density of noradrenaline receptors (Chamberlain et al. 2006), and previous studies found that a reduction in noradrenergic transmission, via propranolol, also led to reduced activity in such limbic regions when participants viewed emotional pictures during the functional magnetic resonance imaging (fMRI) scan (Hurlemann et al. 2010). In addition, previous psychological research has suggested that implicit racial bias might be mediated by affective fear responses (Nosek et al. 2011), supporting the hypothesis that the reduction of negative bias after propranolol (Terbeck et al. 2012) might be correlated with differential limbic brain activation patterns, which underly such arousal responses. Thus, the aim of the present study was to investigate the effect of propranolol on neural responses to racial out- versus in-group faces. We predicted that propranolol would decrease activation in the limbic network to black (out-group) versus white faces (in-group), and that, behaviourally, the IAT score would be also be decreased.

## Method

### Participants and design

Forty right-handed healthy individuals (*M*_age_ = 21.93 years; 24 male, 16 female, all white) participated in this research after written informed consent was obtained. Participants were recruited via poster and email advertisements and were initially medically and psychologically screened, including measurement of blood pressure and the recording of an ECG. Participants with any contra-indications to propranolol administration, as well as any history of mental illness (as assessed by the Structural Clinical Interview for DSM-Clinical Version, SCID-IV, First and Gibbon 1997; Beck’s Depression Inventory, Beck et al. 1961; and Eysenck’s Neuroticism and Psychoticism scale, Eysenck, Eysenck et al. 1985), were excluded from the study. The study was approved by an NHS research ethics committee.

The study used a double-blind, placebo-controlled, parallel group design where participants were randomly allocated to receive either placebo or propranolol (40 mg) orally, 1 h before fMRI, to achieve a peak plasma concentration of propranolol (Gilman et al. 1980) during the scan. The fMRI scan lasted 30 min, and tests of implicit racial associations and explicit prejudice were then completed outside the scanner. Heart rate was measured every 30 min using a pulse oximeter. Due to a technical failure, all data for one participant in the placebo group were not being recorded.

### fMRI task

The design of the fMRI task was based on Phelps et al. (2000); we chose this block design task, since it was also used in the original investigation of IAT effect and racial face perception, and made it thus most comparable to the original paradigm. Additionally, we previously also found robust arousal responses to emotional stimuli using a block design (Murphy et al. 2009). Participants viewed unfamiliar black and white male faces in the scanner. The faces were taken from the Stanford University faces database (https://stanforduniversity.qualtrics.com/SE/?SID=SV_aX0ovSkASZR9Py4). These faces were previously rated by over 100 participants in an online web study on the dimensions of attractiveness, stereotypicality, and age. Attractiveness and stereotypicality dimensions were rated using a 7-point scale, while the age dimension used a 10-point scale. We selected a group of 36 black and 36 white faces that did not significantly differ on the ratings of attractiveness, stereotypicality, and age. In this task, nine 18-s blocks of a baseline fixation cross (condition A) were interleaved with eight 18-s blocks consisting of four blocks of white faces (condition B) and four blocks of black faces (condition C). During each task, block participants viewed nine faces, each presented for 1500 ms, and participants were asked to judge the age of the person in the picture (‘Is the person older than 25 years?’, 0 = No or 1 = Yes). The age rating task was analogous to the procedure used by Wheeler and Fiske (2005). Stimuli were presented using E-Prime (Version 1.0) and a cloned projection displayed to participants on an opaque screen located at the head of the scanner bore, which participants viewed using angled mirrors. Participants responded via an MRI-compatible keypad. Stimulus presentation/participant button presses were registered and time-locked to fMRI data using E-Prime. Both response (judgement of age) and reaction times were recorded for 1000 ms and 500-ms inter-stimulus intervals. Four blocks of resting—staring at the central fixation cross—were interleaved and lasted for 18,000 ms each. Faces were presented in random order. Participants responded using a left and right response button. Response time and responses (using the MRI-compatible keypad) were recorded via E-prime software.

### fMRI data acquisition

All imaging data were obtained using a 3T Siemens Tim Trio system. Functional imaging consisted of T_2_*-weighted echo-planar image (EPI) slices [repetition time (TR) = 2000 ms, echo time (TE) = 28 ms, matrix = 192 × 192], with 3.5-mm slice thickness. The first two EPI volumes in each session were discarded to avoid T1 equilibration effects.

### fMRI data analysis

Functional MRI data were pre-processed and analysed using FSL software (Version 4.8, www.fmrib.ox.ac.uk/fsl). Pre-processing included slice acquisition time correction (using Fourier-space time-series phase shifting), within-subject image realignment, non-brain removal spatial normalisation to a standard template (Montreal Neurological Institute, MNI, 152 stereotactic template), using an affine procedure and spatial smoothing using a Gaussian kernel (5 mm full width at half maximum). The time series in each session was high-pass filtered (to a maximum of 0.0125 Hz). Analyses of data from individual participants were computed using the general linear model approach with local autocorrelation correction. For the faces task, the explanatory variables were modelled: ‘black face’, ‘white face’ and ‘fixation’. In addition, temporal derivatives were included in the model as covariates of no interest to increase statistical sensitivity. All variables were modelled by convolving each block with a haemodynamic response function, using a variant of a gamma function (that is, a normalisation of the probability density function of the gamma function) with a standard deviation of 3 s and a mean lag of 6 s. At the group level, data were analysed using a mixed effects analysis. In a whole brain analysis, significant activations were identified using cluster-based thresholding of statistical images with a height threshold of *Z* = 2.5 and a (corrected) spatial extent threshold of *p* < 0.05. In addition to our whole brain analysis, we also examined limbic network structures as a priori regions of interest.

### Implicit association test

The IAT (Greenwald et al. 1998) was presented using E-Prime (Version 2.0) software. This test consists of congruent and incongruent blocks. In the congruent trials participants have to categorize positive words with white faces and negative words with black faces. In the incongruent trials, participants have to categorise positive words with black faces and negative words with white faces. There were 60 congruent and 60 incongruent trials (20 of each were classified as practice trials). The implicit association bias is revealed by computing the difference in response times for congruent and incongruent blocks (i.e. if response times for incongruent conditions are greater than for congruent ones, a positive IAT score is produced, indicating that the participant had a more accessible association of positive/white face and negative/black face categorisations than for negative/white face and positive/black face associations). The IAT score is represented as a D-measure (See Greenwald et al. 2003). Specifically, a high value indicates strong greater association of black with bad and white with good (higher implicit bias). Faces as well as words and design of the task were analogous to the original version developed by Greenwald et al. (1998). The faces for the IAT task were not the same faces as used in the fMRI task.

### Explicit measure

Explicit racial attitudes were measured using two different methods:

- A feeling thermometer (Converse and Presser 1986) was used, an established method to test self-reported out-group attitudes. This is a 10-item scale in which participants express how cold or warm they feel towards a target group (10 = cold, to 100 = warm). The difference between the items ‘feeling for in-group’ and ‘feeling for out-group’ was calculated. A positive score indicated greater negative/cold feeling towards the out-group.
- In a second explicit measure, participants rated their feelings towards in- and out-groups on six 8-point semantic-differential items [for placebo group (alpha) = .87; for propranolol group (alpha) = .92): 1, 0 = cold, 7 = warm; 2, 0 = positive, 7 = negative; 3, 0 = hostile, 7 = friendly; 4, 0 = trusting, 7 = suspicious; 5, 0 = contempt, 7 = respect; 6, *0* = disgust, 7 = admiration (7)]. The items were coded so that a higher score indicated a more negative out-group evaluation; items 2 and 4 were reverse coded. This method for assessing explicit prejudice towards other groups was based on previous research, for example by Wright et al. (1997).

### Self-reported mood: visual analogue scales

Self-reported mood was assessed using visual analogue scales. Participants indicated on 20-cm lines the degree to which they felt tense, sad, angry, tired, happy or alert. We subsequently measured (in cm) where participants made their cross on the line (0.00–20.00). The higher the score, the greater the indicated level of assessed mood. This measure was obtained at three time points: at baseline (i.e. before the intervention), 1 h after drug/placebo administration, which was just before the scan, and at the end of the experiment (i.e. 150 min after intervention).

## Results

Both groups were well matched across all demographics measured, including depression and personality scores (i.e. there were no significant differences on independent sample *t* tests for all demographic variables; all *p*s *<* .05) (see Table 1).

**Table 1:** Demographic data for placebo and propranolol group

Variable	Mean	SD	*p* value
Gender	PL = 11 female		
	PR = 11 female		
Age	PL = 21.94	PL = 2.84	n.s.
	PR = 21.38	PR = 2.97	
Depression inventory	PL = 1.39	PL = 1.69	n.s.
	PR = .95	PR = 1.40	
Neuroticism scale	PL = 1.56	PL = 1.58	n.s.
	PR = 1.90	PR = 2.02	

*PL* placebo, *PR* propranolol

### Heart rate analysis

We computed a 2 (intervention: placebo versus propranolol)×6 (time) mixed model analysis of variance (ANOVA) with repeated measures on the latter factor. The data from one participant was missing, as the pulse oximeter did not record the data in the fMRI scanner. A significant intervention × time interaction was found [*F*(5,32) = 2.96, *p* < .03; *η*^2^ = .32]. Heart rate was significantly reduced in the propranolol group 60 min after treatment and remained lower for all further 30-min measurement points [at 0 min: propranolol (PR), *M* = 68.57, SD = 10.21; placebo (PL), *M* = 72.71, SD: 9.77; *t*(36) = 1.27, *p* = .21; at 30 min: PR, *M* = 64.67, SD = 9.41; PL, *M* = 70.65, SD = 9.94; *t*(36) = 1.90, *p* = .07; at 60 min: PR, *M* = 59.81, SD = 9.00; PL, *M* = 70.82, SD = 10.38; *t*(36) = 3.51, *p* < .01; at 90 min: PR, *M* = 55.05, SD = 8.78; PL, *M* = 67.94, SD = 9.31; *t*(36) = 4.38, *p* < .00; at 120 min: PR, *M* = 55.90, SD = 8.71; PL: *M* = 69.28, SD = 10.39; *t*(36) = 4.32, *p* < .00; at 150 min: PR, *M* = 56.95, SD = 7.75; PL, *M* = 68.76, SD = 7.63; *t*(36) = 4.71, *p* < .00).

### Self-reported mood

There were no group differences in self-reported mood at any of the three measurement times (all *p*s > .2).

### Implicit association test

**Fig. 1:**
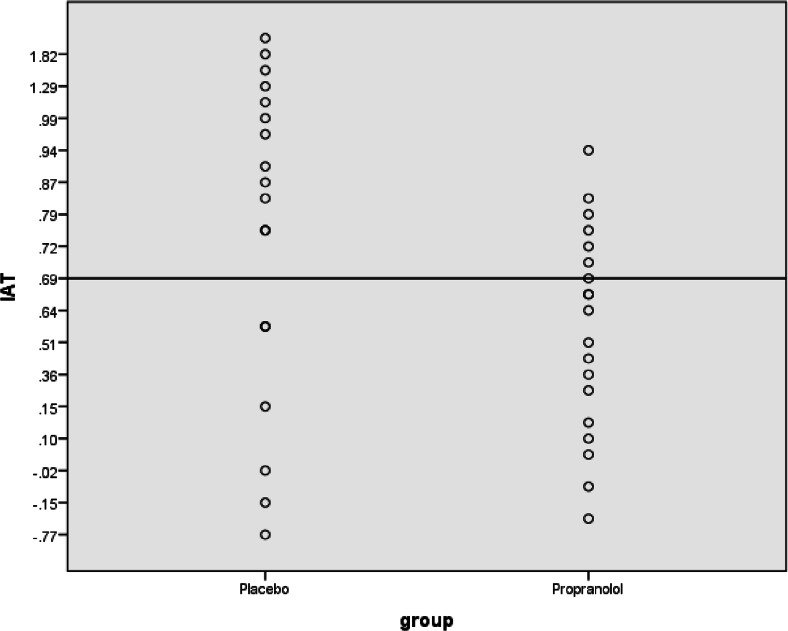
IAT score in placebo and propranolol groups. Scores are normalised by division through the pooled standard deviation for correct trials, as stated in the improved algorithm analysis

The IAT was analysed according to the Greenwald et al. (2003) improved algorithm. In the analysis, all trials were considered. Following the procedures of the improved algorithm, errors were replaced with the mean for correct trials in that block + 600 ms. The resulting values were divided by their pooled standard deviation for correct trials (for a full description of the method, see Greenwald et al. 2003). Three participants had excessive error rates as well as more than three consecutive errors in the main blocks and were thus excluded. In addition, the practice trials of two participants were not included in the main analysis as they were excessively slow in responding (i.e. response times over 4000 ms) during the practice session. The corrected difference between congruent and incongruent trials revealed the standardised IAT effect. The IAT was lower in the propranolol group (*M* = .46, SD = .37) compared to the placebo group (*M* = .81, SD = .75). This difference was significant, one tailed [*t*(35) = 1.83, *p* = 0.04, *d* = .62] (see Fig. 1).

### Explicit measures

We conducted a *t* test to determine group differences in self-reported feelings towards in- and out-groups on the semantic differential measure based on Wright et al. (1997). There were no significant differences in explicit prejudice between propranolol and placebo conditions (*M*_placebo_ = 31.50, SD = 6.00; *M*_propranolol_ = 29.24, SD = 6.35; *t*(37) = 1.14, *p* = .26). For the feeling thermometer (which was non-normally distributed), we conducted a Mann–Whitney *U* test to determine group differences; again, there were no significant differences in explicit racial prejudice (Mrank_placebo_ = 20.78; Mrank_propranolol_ = 19.33; *p* > .2).

### fMRI task and neural responses

In order to determine differences in behavioural responses to the age task (i.e. differences in response and/or response time to black/white faces) following propranolol intervention, we conducted a 2 (intervention: propranolol versus placebo)×2 (race: black face versus white face) mixed ANOVA, with repeated measures on the last factor. We found no interaction effect with intervention (*p* > .05).

**Fig. 2:**
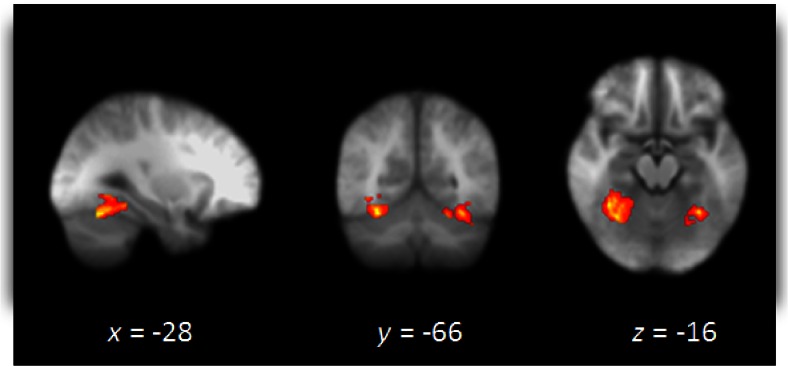
Whole brain image under placebo versus propranolol to black versus white faces. Whole brain image depicting greater activation under placebo versus propranolol to black versus white faces. Image thresholded at *Z* = 2.3, *p* > 0.05, corrected. Images are in radiological format (right brain on *left*)

**Fig. 3:**
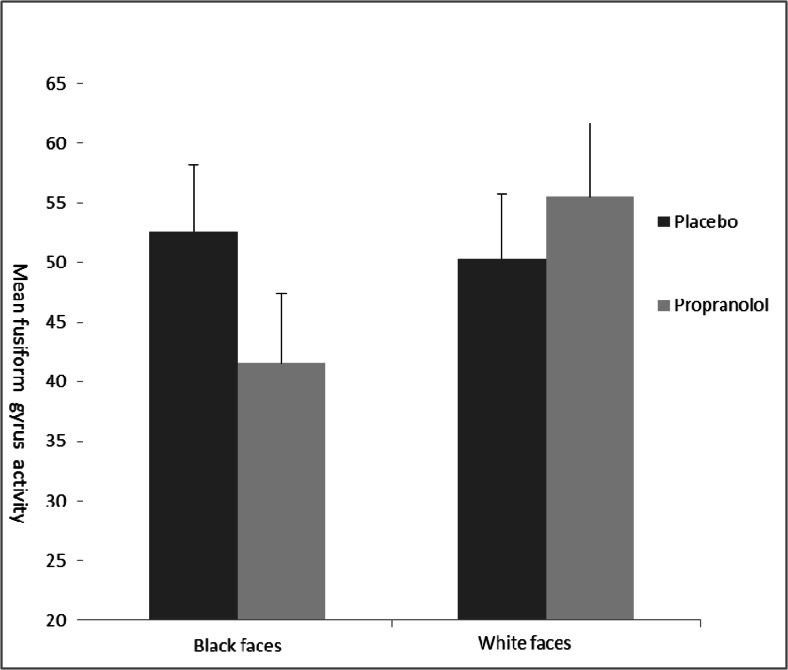
Decomposed interaction in the fusiform gyrus. Mean fusiform gyrus activity in placebo and propranolol group for black faces baseline and white faces baseline

Whole brain analyses revealed greater activation under placebo versus propranolol for black versus white faces in the fusiform gyrus, including the fusiform face area, and the thalamus. Additionally, using an region of interest (ROI) approach generated from the Harvard-Oxford anatomical atlas we observed greater activation under placebo to black versus white faces in the fusiform gyrus bilaterally (Table 2 and Fig. 2). There were no regions that showed greater activation to black versus white faces in the propranolol group as compared to placebo. In addition, for no participant was there greater activation when comparing white versus black faces. ROI analysis in the amygdala confirmed no differential activation of black versus white faces. Importantly, decomposing the significant fusiform gyrus interaction, we found reduced activity to black faces versus baseline as the only effect and only for the propranolol group (Fig. 3).

**Table 2:** Regions of increased activation under placebo versus propranolol for the orthogonal contrast black versus white faces

Brain region	Cluster size (voxels)	Z score	*p* value	*x*	*y*	*z*
Thalamus	3871	4.33	<.001	−18	−30	6
Fusiform gyrus	590	3.57	0.03	−28	−66	−16

Coordinates refer to the position (*x*, *y* and *z* mm) for the peak voxel in each cluster according to the MNI template

**Fig. 4:**
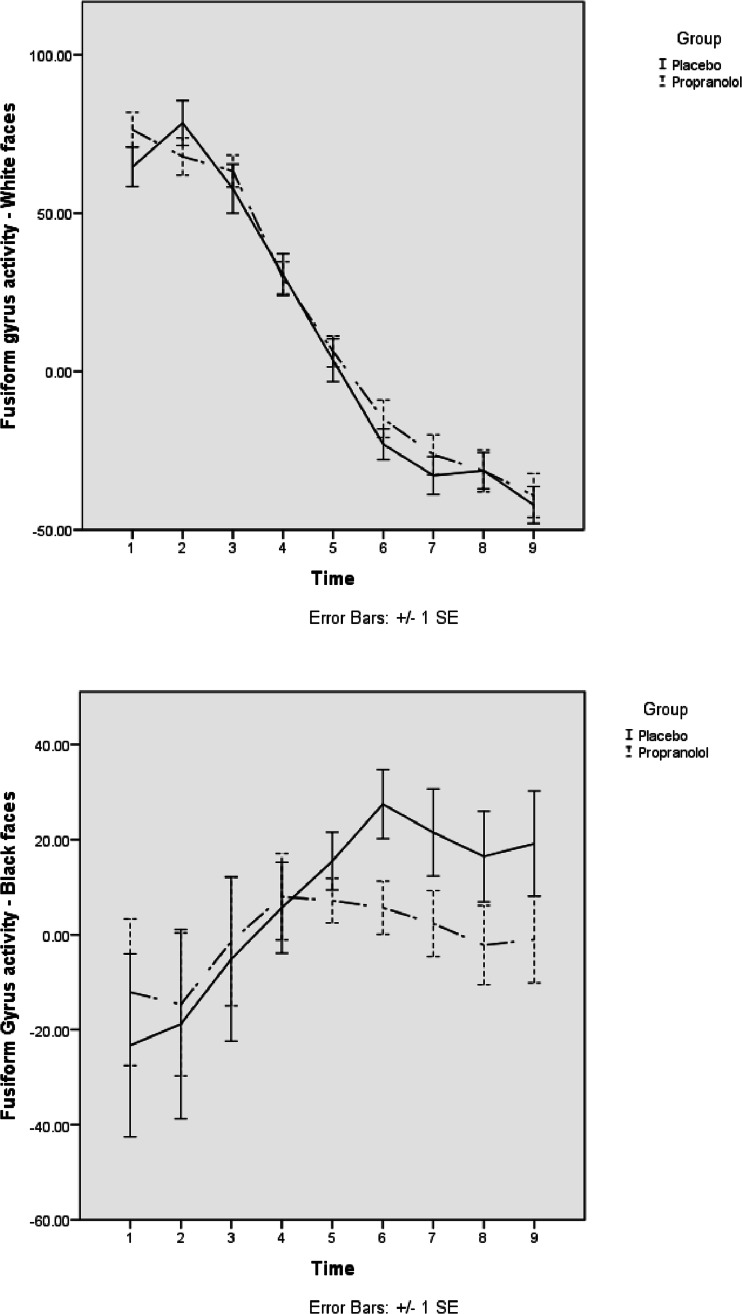
Time course of fusiform gyrus activity for black and white faces in placebo and propranolol group

Additionally, following Natu et al. (2011), we further investigated the time course of activity in the fusiform gyrus during the block. Therefore, nine time points (early–late) were calculated as means for all blocks. The activity was then normalised, and activity for black and white faces was subtracted from baseline activity. Figure 4 shows the time course of fusiform activity for black and white faces within propranolol and placebo group. As depicted in Fig. 4, for placebo as well as propranolol conditions, activity in response to white faces was highest at initial presentation and then declined. For black faces, however, participants in the placebo group showed an opposite pattern where fusiform gyrus activity increased over time. The latter effect was less pronounced following propranolol.

## Discussion

We found that propranolol reduced neural activation to black versus white faces in the fusiform gyrus and thalamus. Propranolol lowered IAT scores, which was significant on a one-tailed test, in line with the same finding we reported in Terbeck et al. (2012), while the measure of explicit prejudice was not affected (again replicating Terbeck et al. 2012). These results extend previous work implicating the fusiform gyrus as part of a network of regions subserving out-group perception, and support the proposal that it is a key region involved in the racial categorisation of viewed faces.

The amygdala has long been regarded as a possible neural substrate of implicit racial bias (Wheeler and Fiske 2005). For example, in an early study, Phelps et al. (2000) found a positive correlation between the IAT score and the extent of amygdala activation when white participants viewed unfamiliar black and white faces. Subsequent fMRI studies have generally found greater amygdala responses to black compared to white faces (Cunningham et al. 2004; Wheeler and Fiske 2005). However, in our study, we found no effect in the amygdala indicating greater activation to black and white faces. Methodological differences may help to explain these apparently inconsistent results. For example, Cunningham et al. (2004) showed that the differential amygdala response to black compared to white faces was dependent on the duration of stimulus presentation, with short presentation times (30 ms) producing a substantially greater effect than longer presentations (525 ms). Our own presentation time was 1500 ms; however, Wheeler and Fiske (2005) reported a significant differential amygdala response to black and white faces in a similar paradigm to our own with a presentation time of 2 s. Hence, demonstration of a greater amygdala response to black versus white faces may depend importantly on experimental parameters such as the nature of the presentation of the visual stimulus and the number of repetitions, as well as the experiences of the participants. Indeed, a recent review of neuroimaging studies and racial prejudice suggests that amygdala activity might not reflect a racial out-group bias per se (Chekroud et al. 2014).

While the amygdala plays an important role in the emotional modulation of the processing of faces, the fusiform gyrus has been strongly linked to the determination of the identity of faces. We did not identify the fusiform gyrus as an a priori ROI for our study. However, bilateral fusiform effects were identified by the whole brain analysis, which supports the post hoc analysis. In addition, there is strong evidence from other studies that the fusiform gyrus, particularly the fusiform face area (FFA), plays a key role in the representation of racial identity (Brosch et al. 2012; Contreras et al. 2013; Golby et al. 2001; Natu et al. 2011).

A study by Contreras et al. (2013) used multivoxel pattern analysis to decode activity in the FFA while participants viewed images of faces of different races and genders. They found that the FFA distinguished both gender and race of individual faces. However, patterns extracted from other face-selective cortical regions involved in early visual processing did not identify either gender or race, suggesting these categorisations might be confined to the FFA.

Relevant to our study, the ability of the FFA to represent race appears to be modified by racial bias as measured by the IAT. For example, Brosch et al. (2013), using a similar multivoxel pattern approach to that of Contreras et al. (2013), found that successful decoding of racial identification in the FFA was only possible in participants with high levels of implicit racial bias. However, and in contrast to Contreras et al. (2013), these authors also found that racial identity could be successfully decoded on the basis of activation patterns in early visual processing areas. They concluded that, while the latter regions distinguished race by identifying basic perceptual features, the FFA was involved in higher order facial identification such as social categorisation.

In our study, propranolol lowered implicit bias and significantly diminished the activation elicited by viewing of black faces in the fusiform area but did not alter the activation to white faces. Natu et al. (2011) presented evidence showing that the processing of black and white faces in white participants has a differential time course in the fusiform area with ‘same race’ faces producing a large early activation, while ‘other race’ faces produce a more delayed response that eventually equals or even exceeds that occurring in response to ‘same race’ faces. Our findings regarding the time course of fusiform gyrus activity support this notion; in both groups, activity in response to white faces was high initially and then declined. In the placebo condition, however, fusiform gyrus activity for black faces increased over time, suggesting that propranolol selectively reduced the differential processing of out-group faces in the fusiform area. Therefore, propranolol may interfere with the ‘other race’ distinction that seems to be an important property of fusiform processing during the recognition of racial identity.

If the fusiform area is a key region for recognising the identity of out-group faces, it may also play an important role in generating implicit racial bias. While the amygdala is a candidate region for this effect, a study by Phelps et al. (2003) indicated that implicit bias measured by the IAT was still present in a participant with bilateral amygdala damage, suggesting that the amygdala itself may not play a critical role in the manifestation of implicit bias. The site of action of propranolol to influence fusiform activity when viewing black faces is not clear from the present study, also since we did not find an overall correlation of IAT score and fusiform gyrus activity. Propranolol is known to influence the amygdala response to negative emotional faces but, as noted above, the current study does not suggest an effect mediated via the amygdala. However, Norbury et al. (2007) found that short-term intervention with the noradrenaline re-uptake inhibitor reboxetine increased responses in the fusiform gyrus to happy faces, suggesting that noradrenaline can modulate responses to emotional faces in this area and supporting the view that the fusiform plays a role in different kinds of complex social categorisation (Bosch et al. 2013). Future research might investigate which specific aspect of racial face perception (e.g. configural or featural processing) might be affected.

We also found reduced activation to black versus white faces in the thalamus; this effect was not predicted but was apparent at the level of whole brain analysis. The thalamus has also been implicated in face perception (Phelps and LeDoux 2005; Vuilleumier and Pourtois 2007); thus, a direct pathway from thalamus to amygdala facilitates the rapid detection of emotionally salient stimuli, and projections from amygdala to the fusiform gyrus have been implicated in the increased activity in the fusiform face area in response to emotional faces (Phelps and LeDoux 2005; Vuilleumier and Pourtois 2007). The thalamus itself is known to be activated by demanding visual tasks involving selective attention and visual discrimination (Sabatinelli et al. 2011), and there are reciprocal connections between thalamus and fusiform face area (Clarke et al. 1999). This may well be relevant to our findings of a similar attenuation by propranolol to out-group faces in the thalamus as in the fusiform face area.

In our previous behavioural study, we found that propranolol significantly lowered racial bias as measured by the IAT. Our findings with respect to the effect of propranolol on the IAT in the present study, while similar in nature, were less robust (significant at a one-tailed level only). However, this might be accounted for by the particular experimental conditions, namely that the IAT was carried out after the task in the scanner, which involved repeated viewing of black versus white faces. Future research might test this proposal. Moreover, plasma levels of propranolol would likely have been declining when the participants completed the IAT. Furthermore, we used a between-group design, as it would be very difficult to have participants performing the same tasks (e.g. IAT and fMRI) twice without affecting the reliability of the results.

In summary, we found reduced activation in both fusiform gyrus and thalamus in participants who received propranolol and who viewed unfamiliar black versus white faces. In addition, the IAT score was lower in participants receiving propranolol. These results support the conjecture that implicit aspects of racial attitudes have an underlying neural component in which face processing in the fusiform gyrus plays an important role. It should be noted, however, the we did not use a functional face localiser to delineate the FFA, which is a weakness of the study. Future work could also link current work on multivoxel pattern analysis in the FFA with the effects of pharmacological manipulation of implicit bias (Terbeck et al. 2012; Contreras et al. 2013). For example, it would be of great interest to determine if propranolol was able to impair the decoding of the FFA activation patterns that represent different racial faces. This could provide a plausible neurobiological substrate for the ability of propranolol to diminish implicit racial bias.
